# On the Association between Gastrointestinal Symptoms and Extragastric Manifestations

**DOI:** 10.1155/2022/8379579

**Published:** 2022-06-18

**Authors:** G. Naoum, S. L. Markantonis, E. Fanerou, G. Siagkas, F. Petropoulos, E. Zafiris, R. Kousovista, V. Karalis

**Affiliations:** ^1^Internal Medicine Clinic, Athens Medical Center, Distomou Street, Maroussi 15125, Greece; ^2^Department of Pharmacy, School of Health Sciences, National and Kapodistrian University of Athens, Athens 15784, Greece; ^3^Department of Mathematics and Applied Mathematics, University of Crete, Heraklion, Crete 71500, Greece

## Abstract

The aim of this study was to investigate the relationship between gastrointestinal (GI) symptoms and extragastric manifestations such as headache, fatigue, and dizziness. A prospective cohort study was conducted in a tertiary hospital in Athens, where patients with GI problems and extragastric symptoms were treated only for their GI problems, and improvement in extragastric manifestations was recorded. Inclusion criteria were an age older than 18 years, the presence of at least one of the three extragastric symptoms investigated in this study (headache, dizziness, and fatigue), and the concomitant presence of at least one gastrointestinal symptom (e.g., nausea, belching, abdominal tenderness, epigastric pain, halitosis, flatulence, diarrhea, bad odor of flatulence, flatulence, and constipation). A standardized questionnaire was used to collect demographic data (such as age, weight, and height), patients' symptoms, laboratory findings (gastric biopsy, gastroscopy, and colonoscopy), and intensity/frequency of GI and extragastric symptoms. Statistically significant associations were found between GI symptoms (nausea, constipation, halitosis, and belching) and dizziness, fatigue, and headache (frequency, intensity, and duration). Treatment of GI problems resulted in a significant improvement in extragastric symptoms within one month of treatment initiation. It should be emphasized that the actual reason for the improvement in extragastric symptoms was solely the resolution of the GI problems, as patients did not receive specific treatments for headache, dizziness or fatigue, or other changes in daily life. This study demonstrates the association between extragastric manifestations and GI disorders.

## 1. Introduction

The gastrointestinal tract (GI) is one of the most basic anatomical systems of the body. Its main function is the continuous supply of water, electrolytes, and nutrients to the body, as well as the elimination of unabsorbed contents [1].

The traditional Persian physicians, Avicenna and Rhazes, believed in a type of headache caused by gastrointestinal problems [2]. In recent decades, a number of research centers around the world have focused their attention and research on the relationship between diseases or disorders of the digestive tract and extragastric diseases and symptoms [3]. In this context, there may be a relationship between headaches and the gastrointestinal tract [4]. Migraine attacks are often accompanied by nausea and vomiting. Recent research has found that people who have daily gastrointestinal problems, such as reflux, diarrhea, constipation, and nausea, are more likely to have headaches than people who do not [4].

The term “gut-brain axis” refers to a bidirectional interaction between the gastrointestinal system (GI) and the central nervous system (CNS). Several studies have found an association between migraine and GI diseases such as *Helicobacter pylori* infection, irritable bowel syndrome, and celiac disease [5]. The gut system affects several brain functions, including cognition, behavior, and even nociception. The gut-brain axis has been linked to a variety of neurological disorders, including multiple sclerosis, mood and anxiety disorders, Alzheimer's disease, Parkinson's disease, and migraines [5].

A potential pathogenic link has been attributed to *H. pylori* for several extragastric manifestations [6, 7]. The latter include pathological conditions such as vascular disease (including atherosclerosis and ischemic heart disease) and several other conditions (e.g., Sjogren's syndrome, Henoch-Schonlein purpura, autoimmune thyroiditis, Parkinson's disease, and anterior ischemic optic neuropathy), skin diseases (chronic idiopathic urticaria, gyroid alopecia), iron deficiency anemia, delayed menarche, type 2 diabetes, allergic asthma, and psychological problems [8–16]. This supports the hypothesis that some microbes can cause disease even far from the site of infection by affecting various physiological processes [17].

Studies that focused on the association between *H. pylori* and the occurrence of headache have shown that the prevalence of headache or classic migraine was higher in patients infected with *H. pylori* [11, 18–20]. The action of *H. pylori* in some of these diseases is probably through an appropriate immune response of the body, which involves chronic stimulation of receptors and the release of vasoactive substances such as cytokines and prostaglandins. It is certainly a complex phenomenon involving the action of many components such as possible other infections, host genetic and immunological factors, differences in *H. pylori* strains, etc. In addition to *H. pylori* infection, the presence of migraine headache has been clearly associated with upper gastrointestinal tract inflammation due to celiac disease or duodenal ulcer, without specific gastrointestinal symptoms or a specific diagnosis of etiology [12, 14, 21]. From the results of the study of the association between digestive disorders and extragastric symptoms/diseases, it appears that there may be an association between digestive disorders and a number of extragastric diseases whose etiology was previously unknown.

A recent study sought to investigate the role of *H. pylori* infection in migraine headaches with and without aura [22]. The results showed an association between *H. pylori* infection and migraine headaches. These results suggest that specific treatment and eradication of this bacterium could lead to a cure or at least a reduction in the severity and duration of migraine headaches. A double-blind, randomized, controlled clinical trial found that eradication of *H. pylori* may have a beneficial effect on migraine headaches [23].

The aim of this study was to investigate the relationship between GI symptoms and extragastric manifestations such as headache, fatigue, and dizziness. A prospective cohort study was conducted in patients with gastrointestinal problems who also suffered from the above extragastric symptoms. Only GI symptoms were treated and improvement in extragastric manifestations was recorded. In addition, the effectiveness of medical treatment related to GI disorders on the extragastric manifestations was investigated.

## 2. Materials and Methods

### 2.1. Clinical Unit—Data Collection

The study was conducted between December 2017 and April 2021 in a tertiary hospital in Athens. Seventy-five patients aged between 18 and 87 years participated from an internal medicine clinic at Athens Medical Center. The study was approved by the hospital ethics committee (D140647—February 2, 2016), and the entire clinical part of the study was conducted in accordance with the Declaration of Helsinki and the International Conference on Harmonization for Good Clinical Practice. All patients gave informed consent before participating in the study. This trial was part of a larger study to evaluate medical management of GI symptoms and their association with extragastric manifestations.

Patients who met the following criteria participated in the study: age over 18 years, presence of at least one of the three extragastric symptoms evaluated in this study (i.e., headache, dizziness, AND fatigue), and concomitant presence of at least one gastrointestinal symptom (e.g., nausea, belching, abdominal tenderness, epigastric pain, halitosis, bloating, diarrhea, bad odor of flatulence, flatulence, and constipation).

Based on the medical history, laboratory tests, and physical examination performed by physicians, patients with malignancies and end-stage renal disease, those taking immunosuppressive medications, and patients with chronic diseases such as diabetes mellitus, hypertension, cardiovascular disease, osteoarthritis, pancreatitis, and positive Australian antigenic hepatitis were excluded. Additional exclusion criteria were as follows: acute sinusitis, brain aneurysm, brain tumor, stroke, encephalitis, meningitis, intracranial hematoma, glaucoma, depression and/or panic attack, and any other brain injury. None of the patients suffered from *H. pylori infection*, as in these cases, treatment with an antibiotic combination would have been necessary. Therefore, all study participants did not require any other drug treatment apart from medications for migraine/headache treatment such as nonsteroidal anti-inflammatory drugs, triptans, ergot alkaloids, and opioids).

In this study, instead of medications for headache/migraine, the participants received treatment for extragastric and GI symptoms (e.g., proton pump inhibitors, *Η*_2_-histamine antagonists, and antiemetics) at the discretion of the physicians. A questionnaire was used to record demographic data, medical history, GI symptoms, and extragastric manifestations of all eligible patients on day 1, day 15, and one month (day 30) of the study. Thus, all participants were monitored for a period of 30 days.

Because of the COVID-19 pandemic, patient recruitment was difficult, and the study took more time than originally anticipated. No power analysis was performed to estimate sample size, and all available subjects who met eligibility criteria during the study period were enrolled.

### 2.2. Questionnaire—Symptoms

All participants underwent a physical examination, and their past medical history was documented at their first visit to the clinic. A standardized questionnaire was developed to collect as much available information as possible to be used later for statistical analysis. The data recorded included demographic data (such as age, weight, and height), patients' symptoms, and any laboratory findings (gastric biopsy, gastroscopy, and colonoscopy). It should be noted that we did not employ a validated questionnaire from the literature to assess the symptoms because there was no one questionnaire that addressed the wide range of extragastric symptoms associated with gastrointestinal illnesses. However, for the purposes of this investigation, we created a questionnaire focusing on the relationship between GI and extragastric symptoms. To reduce bias and measurement errors, the utilized questionnaire had few questions and choices, was written in plain language, and gave the necessary information.

Patients' nongastrointestinal symptoms, i.e., dizziness, headache/migraine, fatigue, blurred vision, skin rashes, white tongue coating, neck pain, back pain, and dermatopathies, as well as gastrointestinal symptoms like belching, abdominal tenderness, epigastric pain, nausea, diarrhea, constipation, bad odor, and flatulence, were recorded. Belching refers to the act of expelling air from the stomach through the mouth. It usually happens when the stomach distends, or swells, as a result of swallowing too much air. The most prevalent causes include excessive eating or drinking, consumption of carbonated beverages, anxiety, or medical disorders such as gastroesophageal reflux disease (GERD), gastroparesis, and gastritis. Abdominal tenderness is a sort of discomfort that occurs when pressure is applied to the abdomen. Abdominal tenderness can occur for a variety of causes like appendicitis, diverticulitis, irritable bowel syndrome (IBS), sports injuries, and gastritis. Epigastric pain refers to pain or discomfort in the upper abdomen. It frequently occurs in conjunction with other typical digestive symptoms such as heartburn, bloating, and gas. Acid reflux, heartburn, lactose intolerance, hiatal hernia, esophagitis, gastritis, peptic ulcer, and Barrett's esophagus are all potential causes of epigastric pain. Flatulence is a medical term for the expulsion of gas from the digestive tract. Flatulence can be caused by a variety of diseases, including constipation, gastroenteritis, food intolerances such as lactose intolerance, IBS, Crohn's disease, celiac disease, diabetes, gastroesophageal reflux disease (GERD), and peptic ulcers. Finally, bad breath (halitosis) is an umbrella phrase for natural odors emanating from a person's mouth that may come from dental health habits or indicate problems from stomach.

The intensity of symptoms was indicated with numbers on the following scale: 0: no symptom (no intensity), 1: little (low intensity), 2: moderate (moderate intensity), and 3: severe (high intensity). For the purposes of this study, no discrimination was made between headache and migraine. Also, the only intervention was treatment of the GI disorders and no other medications or changes in daily life were made.

### 2.3. Statistical Analysis

All identifiable patient data were transformed into anonymous information by the principal investigator of the study and then statistically analyzed. Statistical distribution of variables was tested by applying normality tests (Shapiro-Wilk, Anderson-Darling). Levene's test was used to test heteroskedasticity. Categorical variables were compared using chi-square analysis, and their measures of association were tested between nominal variables using lambda, Cramer's V, and phi and between ordinal variables using Somer's *d*, Kendall's tau − *c*, and gamma. Depending on the type and characteristics (e.g., normal distribution of the data) of the variables, parametric (one-way analysis of variance (ANOVA)) or nonparametric methods (one-way analysis of variance, Kruskal-Wallis test) were used for comparisons between numerical and categorical variables. When differences between variables were significant, The Tukey and Bonferroni tests were performed to examine possible differences between pairs, as well as the nonparametric Dunn's multiple comparison post hoc test. Comparisons between patients' first and second visits and first and third visits were tested using the nonparametric Wilcoxon signed-rank test. In all analyses, the significance threshold was set at 5%. All statistical analysis was implemented in R version 4.1.1 (https://www.r-project.org).

## 3. Results and Discussion

In the present study, the relationship between extragastric symptoms and digestive disorders was investigated in a group of 75 patients (men and women). The demographic data of the patients are shown in Table 1. Two-thirds (67%) of the patients were women and 33% were men. The mean age was 48.7 years, and the mean weight was 74.4 kg. None of the participants required medication (e.g., NSAIDs, triptans, and ergot alkaloids) for the treatment of extragastric symptoms, and all of them showed significant improvement in both their GI and extragastric manifestations.

A statistical analysis was performed to investigate a possible association between nongastrointestinal symptoms and GI disorders at the patients' first visit (Table 2). A positive association meant that the presence of a GI symptom also led to an increase in extragastric manifestation. In this context, dizziness frequency showed a significant positive association with nausea (*p* = 0.004), as well as with halitosis (*p* = 0.03) and the working conditions (*p* = 0.022) of each patient. A positive association was found between intensity of dizziness and nausea (*p* = 0.023). A positive association was also found between dizziness duration and nausea (p =0.012), while dizziness duration and constipation were negatively associated (*p* = 0.05). There was also a negative association between headache intensity and constipation (*p* = 0.025). For frequency of fatigue (*p* = 0.034), intensity of fatigue (*p* = 0.02), and duration of fatigue (*p* = 0.002), all three variables were positively associated with belching.

**Figure 1:**
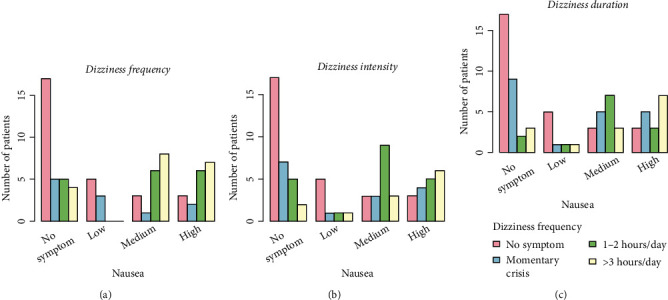
Association between nausea (no, low, medium, and high) and (a) dizziness frequency, (b) dizziness intensity, and (c) dizziness duration at the first visit. The statistically significant associations are listed in Table 2.

**Figure 2:**
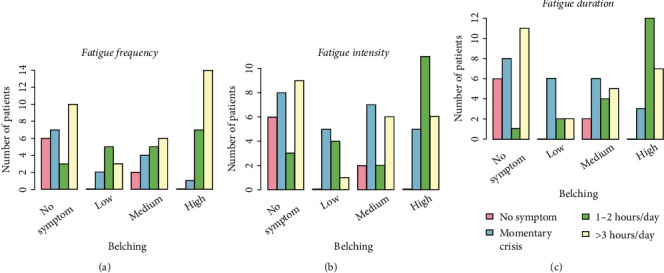
Association between belching (no, low, medium, and high) and (a) fatigue frequency, (b) dizziness intensity, and (c) dizziness duration at the first visit. The statistically significant associations are listed in Table 2.

A graphical representation of the relationships between the reported gastric symptoms and dizziness or fatigue (frequency, intensity, and duration) can be seen in Figures 1 and 2.  Figure 1(a) shows that the majority of patients without nausea symptoms were associated with a low frequency of dizziness. As the intensity of nausea increased, the frequency of dizziness symptoms also increased. Similar results were also observed for dizziness intensity and duration and their association with nausea (Figures 1(b) and 1(c), respectively). Significant associations were found between fatigue and belching (Figure 2). An increase in the severity of belching symptoms was associated with an increase in the proportion of patients with fatigue frequency (Figure 2(a)), intensity (Figure 2(b)), and duration (Figure 2(c)). Since it is confusing to indicate all these significant associations on a graph, the *p* values for statistically significant comparisons are listed in Table 2.

In some cases, associations were found between the extragastric symptoms and the GI disorders. However, these relationships were not statistically significant at the 5% level but at a slightly higher level and were therefore considered trends (Table S1 in the Supplementary Material). Specifically, trends were found for the association between headache duration and abdominal tenderness (*p* = 0.077), dizziness frequency and epigastric tenderness (*p* = 0.075), fatigue frequency and nausea (*p* = 0.073), and bloating (*p* = 0.077). There were negative weak association between dizziness frequency and constipation (*p* = 0.083), dizziness intensity and belching (*p* = 0.065), and dizziness duration and belching (*p* = 0.082). It is worth noting that despite the fact that dizziness (frequency, intensity, and duration) and nausea are positively associated, a negative relationship was found between nausea and dizziness duration (Table 2). This might appear strange since nausea and constipation are usually associated with each other. However, in our study, no relationship was found between nausea and constipation; Spearman's rho coefficient was equal to 0.157 with *p* value = 0.179 indicating no statistically significant correlation between these two variables. Besides, the negative association between constipation and dizziness duration was marginally significant (*p* value = 0.05).

**Figure 3:**
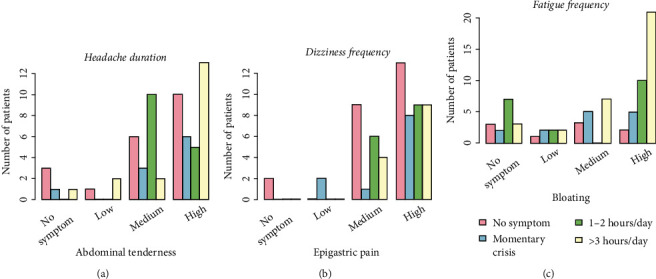
Association between gastrointestinal symptoms and extragastric manifestations. (a) Abdominal tenderness vs. headache duration. (b) Epigastric pain vs. dizziness frequency. (c) Bloating vs. fatigue frequency. The statistically significant associations are listed in Table 2.

A graphical representation of the trends can be found in Figure 3. Visual inspection of Figure 3 clearly shows that the increase in abdominal tenderness (Figure 3(a)), epigastric tenderness (Figure 3(b)), or bloating (Figure 3(c)) led to an increase in the proportion of subjects with extragastric symptoms (either headache duration, dizziness duration, or dizziness frequency).

**Figure 4:**
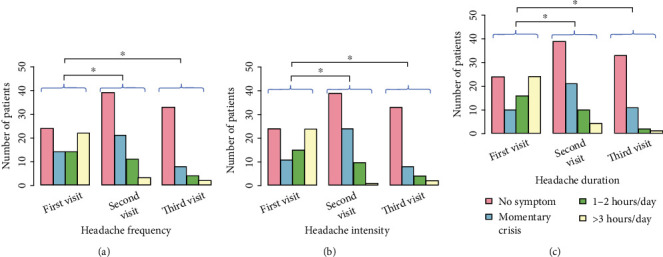
Reduction of headache symptoms from the first visit to the second (two weeks after) and third visits (one month after the 1st visit). (a) Headache frequency. (b) Headache intensity. (c) Headache duration. The asterisk (^∗^) indicates a statistically significant relationship between the marked groups.

**Figure 5:**
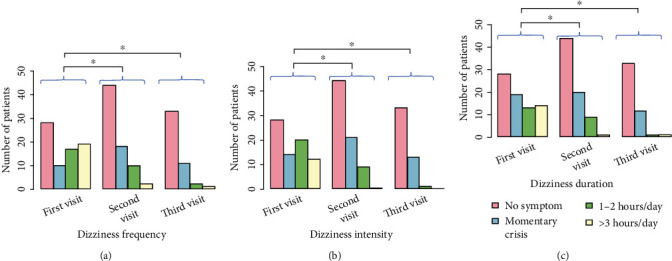
Reduction of dizziness symptoms from the first visit to the second (two weeks after) and third visits (one month after the 1st visit). (a) Dizziness frequency. b) Dizziness intensity. (c) Dizziness duration. The asterisk (^∗^) indicates a statistically significant relationship between the marked groups.

**Figure 6:**
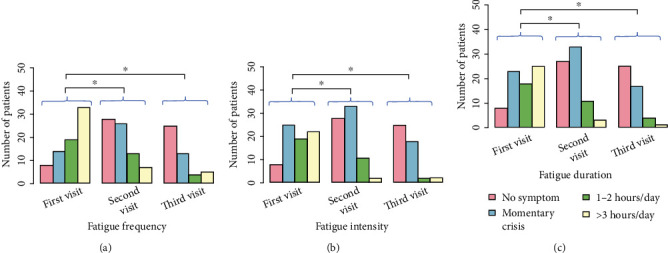
Reduction of fatigue symptoms from the first visit to the second (two weeks after) and third visits (one month after the 1st visit). (a) Fatigue frequency. (b) Fatigue intensity. (c) Fatigue duration. The asterisk (^∗^) indicates a statistically significant relationship between the marked groups.

The reduction of GI symptoms and extragastric manifestations was monitored over a 30-day period after the initial clinic visit. Statistical analysis showed remission of symptoms during treatment. There were statistically significant differences (using the Wilcoxon signed-rank test) in all extragastric symptoms (headache, dizziness, and fatigue) between the first and second clinic visits and between the first and third clinic visits. The reduction of symptoms (frequency, intensity, and duration) for headache, dizziness, and fatigue is shown graphically in Figures 4–6. In these graphs, it can be seen that the proportion of patients with intense extragastric symptoms decreases over time. The statistically significant characteristics (*p* value < 0.001) were the frequency of headache (Figure 4(a)), intensity of headache (Figure 4(b)), duration of headache (Figure 4(c)), frequency of dizziness (Figure 5(a)), intensity of dizziness (Figure 5(b)), duration of dizziness (Figure 5(c)), frequency of fatigue (Figure 6(a)), intensity of fatigue (Figure 6(b)), and duration of fatigue (Figure 6(c)). The *p* values for statistically significant comparisons are presented in Figures 4–6.

### 3.1. Overall Assessment

The current study sought to investigate the relationship between gastrointestinal symptoms and extragastric manifestations such as headache, fatigue, and dizziness. The efficacy of drug treatment of GI problems on extragastric symptoms was also investigated. None of the participants required medication (e.g., NSAIDs, triptans, or ergot alkaloids) for the treatment of extragastric symptoms, and all of them had a considerable improvement in both their gastrointestinal and extragastric manifestations after completing the study and only by the administration of gastrointestinal specific treatment.

Statistically significant associations were found between GI symptoms such as nausea, bad breath, constipation, belching, and the duration/intensity/frequency of extragastric manifestations such as dizziness, fatigue, and headache (Table 2, Figures 1–3). The presence and/or increase in severity of GI symptoms was associated with more frequent and/or more intense extragastric symptoms. Similar associations were observed for other conditions (e.g., constipation and dizziness duration) but were not found to be statistically significant (Table S1). Although these associations were evident from the graphs (Figure 3), no statistically significance was found, likely due to the limited sample size. It is expected that further studies with larger numbers of subjects will provide evidence of these associations. The course of GI symptoms and extragastric manifestations was followed for 30 days after the first clinic visit. The proportion of patients with severe extragastric symptoms was found to decrease over time. Overall, a significant improvement in the severity and frequency of extragastric symptoms was observed in the majority of participants. It should be emphasized that the actual reason for the improvement in extragastric symptoms was solely the resolution of GI problems, as patients did not receive specific treatments for headache, dizziness, or fatigue or other changes in daily life.

The results of this study are consistent with similar findings in the literature. A cross-sectional observational study [24] examined the relationships between microbiome composition and fatigue in patients with advanced cancer. Microbial species with unique compositions were found in cancer patients with high and low fatigue. Another large-scale survey of 1,100 individuals found that two or more of the four symptoms (fatigue, pain, digestive problems, and sleep disturbances) occurred together [25]. Symptoms can occur in a variety of combinations and are strongly associated with health-related quality of life. Patterns associated with fatigue and malaise, as well as those involving multiple symptoms, should be given special consideration.

As for migraine, several studies have found that it is related to GI problems such as diarrhea, constipation, dyspepsia, and gastroesophageal reflux. In addition, several GI diseases such as *H. pylori* infection, irritable bowel syndrome, and celiac disease have been associated with migraine [5]. Another recent study investigated the association between extragastric symptoms and digestive problems in 118 participants [4]. It was clear from the data that there is considerable overlap between migraine and GI symptoms, which has been shown to alter a patient's timing of medication intake due to nausea and fear of vomiting, as well as absorption of oral migraine medications.

Because the brain and GI tract are so closely linked via neurological, endocrine, and immunological pathways, one might expect a link between headache and GI disorders [26]. Several functional and organic GI disorders such as functional dyspepsia, irritable bowel syndrome, gastroesophageal reflux disease, and celiac disease associated with mucosal irritation and/or chronic inflammation are linked to frequent transmission of noxious nerve impulses from the GI tract to the CNS [27]. People who regularly experience GI symptoms had a higher prevalence of headaches, with a stronger association with increasing frequency of headaches [28].

The function of *H. pylori* in migraine manifestations should be particularly emphasized. In this regard, a study investigated the association between *H. pylori* infection, gastroesophageal reflux disease, peptic ulcer, duodenal ulcer, and migraine in patients undergoing upper GI endoscopy for refractory dyspepsia [29]. Migraine has been associated with GERD, *H. pylori* infection, and DU, and treatment of the underlying GI disease may help reduce migraine. Another population-based study discovered an association between migraine and gastrointestinal dysfunction such as irritable bowel syndrome, reflux, and dyspepsia [30].

It is worth noting that the composition of the gut microbiota plays an important role in the gut-brain axis. This occurs through two mechanisms: (a) indirect signaling, which includes microbiota-derived neurotransmitters, pro-inflammatory chemicals, and hormones, and (b) a direct link. Changes in the profile of the gut microbiota can result from psychological and physical stressors. These stressors increase the production of corticotrophin-releasing hormone in the hypothalamus, which stimulates adrenal cortisol release and can cause changes in gut permeability by altering the microbiota profile [5].

Dietary methods, probiotic supplements, and vitamin D3 administration are advocated as nonmedical therapeutic strategies [5]. Many dietary approaches have been proposed for migraine patients; however, it is currently unknown whether diet can be used to improve migraine treatment [5]. Probiotic supplements may help reduce migraine attacks. Some research has looked at the effects of administering probiotics on migraine headaches. A randomized, controlled, double-blind trial examined the efficacy of daily administration of a probiotic mixture of 14 strains or a placebo for 8 weeks in chronic migraineurs and 10 weeks in episodic migraineurs. Although blood levels of selected inflammatory biomarkers did not change significantly, probiotic therapy resulted in significant improvements in migraine frequency and intensity and abortive medication use in the cohort studied [5]. The composition of the gut microbiota was altered by treatment with vitamin D3. Supplementation of healthy individuals with vitamin D3 for 8 weeks also reduced the number of *Helicobacter* sp. Administration of vitamin D has been shown to reduce the severity and frequency of migraine attacks [5]. Obesity has been associated with an increased risk of episodic and chronic migraine, while weight loss has been associated with a reduction in the intensity, frequency, and duration of migraine headaches in adults and adolescents [5].

A limitation of this study was the small sample size of 75 patients, which prevented the identification of further relationships between GI symptoms and extragastric manifestations. More powerful clinical trials would increase the possibility of finding further associations and strengthen the findings of this study. In addition, randomized clinical trials in which patients are divided into groups, those receiving treatment for the GI problems and a control group, would provide more evidence. In addition, studies stratified by age, gender, symptom severity, and medication would have greater discriminatory power. It is also worth mentioning that certain people are more prone to reporting symptoms from all parts of the body, which has nothing to do with an underlying functional bowel issue but may indicate some type of autism spectrum disorder (ASD). Apart from the typical manifestation of ASD, namely, how a person perceives and socializes with others, ASD has been connected to higher awareness and better documenting of behaviors [31]. Furthermore, it is now widely acknowledged that people with ASD are more susceptible to pain [32].

## 4. Conclusions

This study investigated the association between GI symptoms and extragastric manifestations (such as headache, fatigue, and dizziness). Statistically significant associations were found between GI symptoms (nausea, constipation, halitosis, and belching) and dizziness, fatigue, and headache (frequency, intensity, and duration). Dizziness manifestations have been found to be positively associated with nausea and halitosis, while all aspects of fatigue were positively related to belching. The opposite was observed for constipation, which negatively affected dizziness duration and headache intensity. This study also showed that treatment of GI problems resulted in significant improvement of extragastric symptoms within one month after the start of treatment. However, the fact that the present study was multifactorial and based on only a small sample of patients made it difficult to draw statistically significant conclusions for some other associations. Overall, this study demonstrates the association between extragastric manifestations and GI disorders. Further prospective studies with a large number of subjects should be conducted to prove this association.

## Figures and Tables

**Table 1:** Demographic characteristics of the patients enrolled in this study.

Characteristic	Mean value^∗^^,^^∗∗^
Sample size	75
Gender	
Male	33% (25)
Female	67% (50)
Age (years)	48.7 ± 16.9
Weight (kg)	74.4 ± 18.1
Height (cm)	168 ± 9.85
Body mass index (kg/m^2^)	26.0 ± 5.1

^∗^Values in the parentheses refer to the number of patients. ^∗∗^The ± values refer to the estimated standard deviation.

**Table 2:** Statistically significant associations between gastrointestinal (GI) symptoms and extragastric manifestations. The observed significance levels (*p* values) are reported along with the Goodman and Kruskal's gamma values which refer to the rank of association. Gamma values higher than zero indicate positive association and vice versa.

GI symptom	Extragastric symptom	Goodman and Kruskal's gamma value	*p* value
Nausea	Dizziness frequency	0.472	0.004
Halitosis	Dizziness frequency	0.229	0.03
Nausea	Dizziness intensity	0.473	0.023
Nausea	Dizziness duration	0.474	0.012
Constipation	Dizziness duration	-0.233	0.05
Constipation	Headache intensity	-0.219	0.025
Belching	Fatigue frequency	0.376	0.034
Belching	Fatigue intensity	0.208	0.02
Belching	Fatigue duration	0.221	0.002

## Data Availability

Data is available upon request from the author G.N.

## Supplementary Materials

Supplementary Materials Table S1: a tabular list of associations between gastrointestinal (GI) symptoms and extragastric manifestations, which were not significant, but close to the theoretical type I error value of 5%, and therefore were considered “trends.”
