# Supplementary material for: On the Association between Gastrointestinal Symptoms and Extragastric Manifestations
Source: Gastroenterol Res Pract. 2022 Jun 18;2022:8379579. doi: 10.1155/2022/8379579 (PMC9233578; doi:10.1155/2022/8379579)
Supplement: Supplementary Materials — Table S1: a tabular list of associations between gastrointestinal (GI) symptoms and extragastric manifestations, which were not significant, but close to the theoretical type I error value of 5%, and therefore were considered “trends.” [file 8379579.f1.docx]

**Supplementary Material**

**Table S1.** Associations between gastrointestinal (GI) symptoms and extragastric manifestations which were not significant but close to the theoretical type I error value of 5% and therefore were considered as “trends”. The observed significance levels (p – values) are reported along with the Goodman and Kruskal’s gamma values which refer to the rank of correlation. Gamma value higher than zero indicate positive correlation and vice versa.

| **Extragastric symptoms** | **Characteristic 2** | **Goodman and Kruskal’s gamma value** | **p-value** |
| --- | --- | --- | --- |
| Headache duration | Abdomen sensitivity | 0.170 | 0.077 |
| Dizziness Frequency | Epigastric sensitivity | 0.184 | 0.075 |
| Fatigue frequency | Nausea | 0.297 | 0.073 |
| Fatigue frequency | Bloating | 0.336 | 0.077 |
| Dizziness frequency | Constipation | -0.215 | 0.083 |
| Dizziness intensity | Eructation | 0.036 | 0.065 |
| Dizziness duration | Eructation | -0.003 | 0.082 |
